# Supplementary material for: Assessment of Food and Waterborne Viral Outbreaks by Using Field Epidemiologic, Modern Laboratory and Statistical Methods—Lessons Learnt from Seven Major Norovirus Outbreaks in Finland
Source: Pathogens. 2021 Dec 14;10(12):1624. doi: 10.3390/pathogens10121624 (PMC8707936; doi:10.3390/pathogens10121624)
Supplement: Supplementary file 1 [file pathogens-10-01624-s001.zip › Supplementary file 1.pdf]

Outbreak_1:

A norovirus outbreak among the participants of 75-year birthday party in Tampere

The local food authorities were informed about gastrointestinal illness among participants of a birthday party on Tuesday 27.3.2018. The outbreak investigations were initiated immediately and the implicated food service was inspected on 29.3.2018. The on-line cohort study for the participants of the birthday party was opened on the following day. The local infection control team organized the interviews about the symptoms with the symptomatic persons and fecal sampling.

According to the cohort study, 12 persons fulfilled the case definition. The typical symptoms were diarrhea, vomiting, fever, nausea and stomach ache. All those fallen ill recovered by 16.4.2018 (time of the final internal information release to the participants).

The cocktail snacks for the birthday party were ordered from a food service. The likely vehicle was smoked cheese mini-burgers which were prepared in the food service. According to the cohort study, eating the mini-burgers increased the risk of illness to 5.00 times (95 % confidence intervals 1.58-29.81), other cocktail foods were not significant. The mini burgers were most likely contaminated during preparation from the kitchen surfaces or by the hands of the kitchen employer.

Some of the staff members of the food service had had gastrointestinal symptoms already before the preparation of the birthday party snacks took place, but all had followed the national guidelines of withdrawal from risk food practices for two days after the illness. In addition to party participants, some of the staff fell ill at the same time, presumably due to consumption of the same mini-burgers. Altogether 10 fecal samples were obtained from the ill birthday party participants and staff members. Of those, seven were positive for norovirus, genogroup II.

The likely cause of this outbreak was mini-burgers, contaminated by staff preparing those.

Epidemic curve

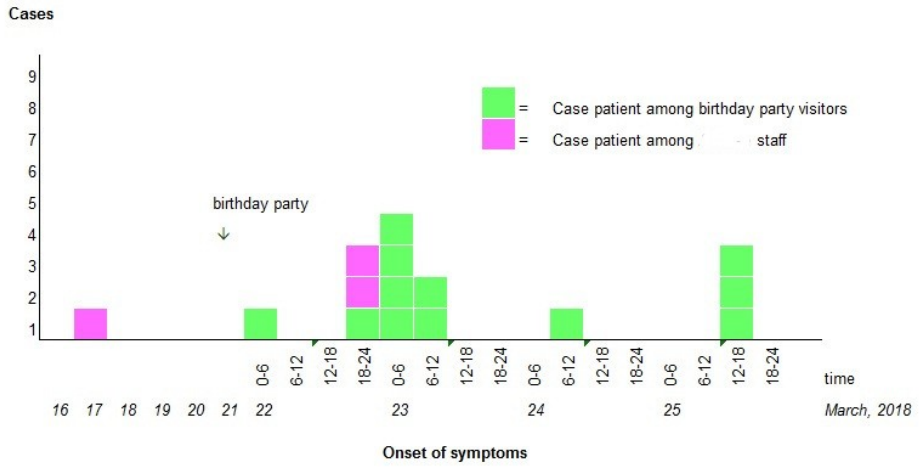

Outbreak_2:

The outbreak investigation from the Kaleva high school is finalized (modified final press release)

The likely cause of the norovirus outbreak in Kaleva high school was cheese rolls prepared in the institutional kitchen of the school. Norovirus outbreaks are common food and waterborne gastrointestinal outbreaks. Norovirus infections are also easily transmitted from person-to-person.

The local food authority was informed of the norovirus outbreak in Kaleva high school from the local newspaper website on 9.2. around noon. The outbreak control actions were initiated immediately and the first outbreak press release was given on 9.2. at 14.

It was observed very soon that cases were occurring especially among third year students, who had participated in mathematical preliminary exam. Altogether 53 suspect cases were notified, of which 44 were third year students. The food served during the 6-hour mathematical exam were cheese roll, squash and a yogurt. The cheese roll was considered the most likely vehicle as it was found out that three staff members who had eaten the left over cheese rolls had also fallen ill. Additionally five other students fell ill who had likely eaten a roll from the school canteen. Additionally one of the preliminary exam participant had taken the cheese roll home and a household member became ill after consuming the roll. The common ingredient between canteen and exam rolls was cucumber.

Altogether 83 third-year students participated to the epidemiological cohort study, of which 82 were enrolled (one traveling abroad was excluded). Of those participating, 72 % (59/82) had taken part in the mathematical preliminary exam. The main symptoms were diarrhea 14 % (11/80), vomiting 23 % (19/82) and fever 20 % (16/80). Common general symptoms were nausea 33 % (27/81) and stomach ache 28 % (23/82). Also one case of bloody diarrhea was observed. Participating in the mathematical preliminary exam increased the risk of illness to 2.4-fold and eating the cheese roll in the exam 6.1-fold.

Norovirus spread from the cheese and cucumber filled rolls prepared in the central school kitchen

Norovirus was detected from feces from altogether five ill third year students and three staff members, of which one was kitchen staff. Norovirus analysis was done also for the cucumber slices in the rolls. No norovirus was detected which may be due to small amount of norovirus in the roll samples. This is a common finding, especially if the virus has been contaminated through hands. Also the kitchen surfaces and environment were negative for norovirus, but the samples were taken only a week after the incident by which time all environment and surfaces had been cleaned and disinfected several times.

The most likely vehicle for the Kaleva high school norovirus outbreak was cheese rolls, prepared in the school kitchen. Additionally a few persons had eaten other rolls containing the same cucumber from the school canteen. The rolls were most likely contaminated during preparation either by kitchen surfaces or the employer's hands. For the future exams, own food is recommended to decentralize the risk.

Additional information

Sirpa Räsänen, City epidemiologist, patient investigations, tel. 040 123 4567

Katri Jalava, City official veterinarian, trace back and food investigations, tel. 040 891 0111

Outbreak_3:

Norovirus outbreak caused by contaminated strawberries among two groups of customers of a local food service in Western Finland

The local food authority was notified of a suspect gastrointestinal outbreak on 7.3.2017 among altogether 62 persons (group A) evening party on 4.3. The foods served had been prepared by a local food service C. This food service C had also delivered a strawberry, toffee and a sandwich cake to birthday party B. Altogether 23 persons participated in Birthday Party B, 16 guests on Saturday 4.3. and 5 on Sunday 5.3. Gastrointestinal illness was reported also from Birthday Party B.

The food control inspected the food service C on Wednesday 8.3.2017. During the inspections, the menus, preparation and ingredients, also in detail the cakes, routine practices in the kitchen and hygiene were checked. From the participants of the evening party, some left over foods were obtained, ingredients were not available. One of the foods in evening party was salad with fresh strawberries (packed in EU). From the importer information was obtained that those strawberries were from Morocco. Same strawberries were used for decorating the Birthday Party B cakes. There were no other food stuff in common between Evening Party A and Birthday Party B. Fecal samples were requested from patients and two kitchen staff. Food samples were taken from the green salad (with strawberries) and strawberry cake (there were no decorative strawberries left). The cohort study was directed to both A and B groups about the foods consumed and symptoms.

Altogether 86 % (72/84) responded to the study, of these 68 % (49/72) fulfilled the case definition (diarrhea, vomiting or at least two other symptoms). Main symptoms were diarrhea 61 % (44/72) and vomiting 49 % (35/71), also general symptoms of nausea 57 % (41/72) and stomach ache 49 % (35/72) were recorded. Case patients also had fever 17 % (12/72) and bloody diarrhea 1 % (1/68). The questionnaires were combined with respect to strawberries. If a person was exposed to strawberries, the risk of illness increased to 2.3 fold (95 % confidence intervals 1.2-6.8) compared to those non-exposed. Other food items were not significant for the Party A. The fecal samples from kitchen staff were negative for viral pathogens. As an additional finding, fecal indicator bacteria were detected from the well of the location of the Party A but this was considered not to be related to the outbreak. The well water was negative for viruses and hygienic measures were taken.

Altogether 16 party A and B ill persons had norovirus genogroup II detected in their fecal samples. From the salad residues and its strawberry piece, norovirus genogroup II was also detected. There was too little genomic material for further sequencing of the sample. The implicated strawberries have been delivered elsewhere with no illness reports. The strawberries were sold with marking ”EU”, the original package was not available. According to importer the strawberries were Moroccan. The strawberries were washed in the kitchen premises, also a potential source of contamination. This outbreak was caused by norovirus contaminated strawberries. Also heating recommendation should be considered for fresh strawberries if similar outbreaks start to occur more commonly.

Additional information: Official veterinarian, katri.jalava@email.fi, 040 891 0111

Epidemic curve. Cases by the onset of symptoms among group A and group B participants.

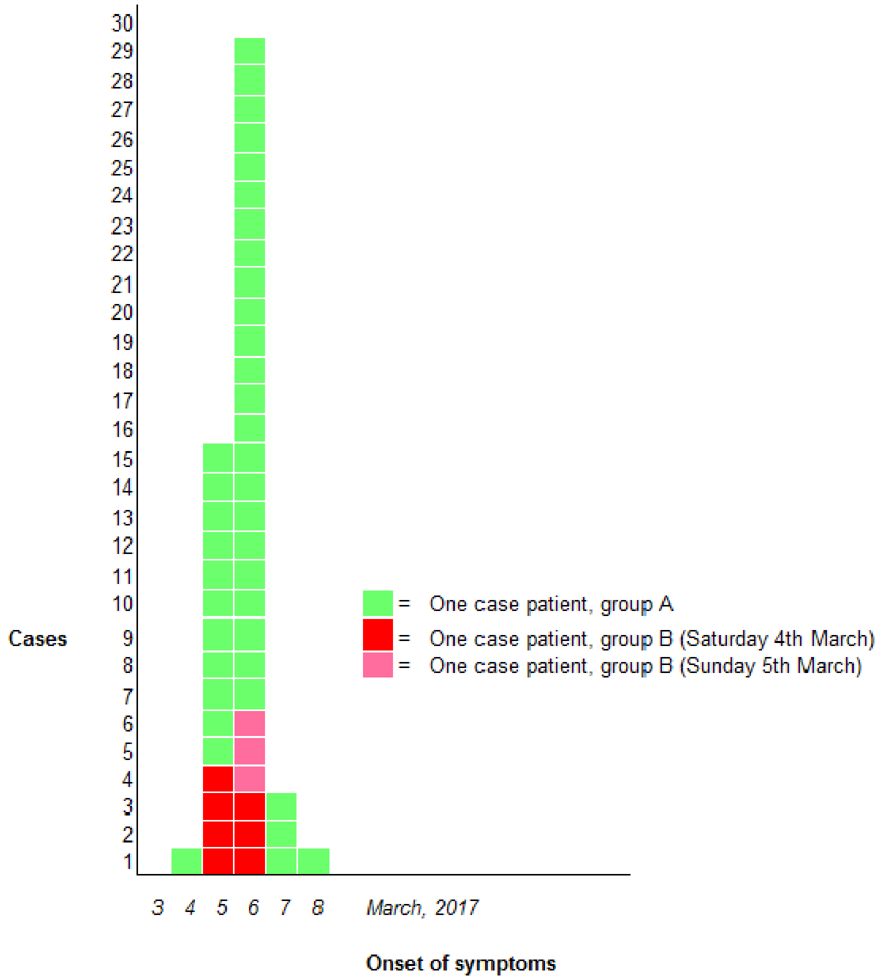

Outbreak_7:

Outbreak information release to the participants of the Christening party and those responding to the questionnaire study

A Christening party was held in the local parish house on 22.10.2017 with 38 participants. Of those, 22 fell ill between 23. and 24.10.2017 with acute gastroenteritis, also secondary cases within households were detected. The foods served in this function included both salty and sweet foods. The foods were prepared by a local food service and also within the household of one private individual. An inspection to the local food service and parish house was carried out, no major violations leading to an outbreak could be detected. Water was obtained from the local water network, no construction work had been carried out.

Fecal samples were requested from those ill and kitchen staff, and one secondary household case. A norovirus, genogroup II was detected. Based on the analytical cohort study, no food could be implicated as a likely source of the outbreak.

Norovirus infection is highly transmissible and may occur by person-to-person transmission in an effective way. Based on the outbreak investigation, the most likely mode of transmission in this outbreak was a person-to-person spread.

More information: Official veterinarian Katri Jalava, katri.jalava@email.fi 040 891 0111
