# Supplementary material for: Assessment of Food and Waterborne Viral Outbreaks by Using Field Epidemiologic, Modern Laboratory and Statistical Methods—Lessons Learnt from Seven Major Norovirus Outbreaks in Finland
Source: Pathogens. 2021 Dec 14;10(12):1624. doi: 10.3390/pathogens10121624 (PMC8707936; doi:10.3390/pathogens10121624)
Supplement: Supplementary file 1 [file pathogens-10-01624-s001.zip › Supplementary file 3.pdf]

How to create an electronic questionnaire

- There are several different electronic reporting forms that may be used. Your unit may have its own that you would like to use.
- An example with free Kobo system (supported by several UN and other international aid organizations, co ordinated by Harvard Humanitarian Initiative)
- The form may be modified to be used for initial symptom inquiry, in-depth interview, or for a cohort study questionnaire (example file)
- The idea is as follow: The questionnaire is being created to an Excel spreadsheet, which is uploaded to a website (in the similar manner to attaching a file to your email) through Kobo website. Kobo system then transforms this spreadsheet to a questionnaire, which the participants may fill in on-line. The results will be saved to a separate file, where you may upload it as an excel spreadsheet. These instructions guide you how to create that questionnaire.
- The questionnaire is created to an excel file with three sheets. A model form is being given with the most basic questions included that are needed in a simple cohort study. The form may also be filled in off-line and only submitted once online again.
- The form is easy to use for the end user and it has a pleasant outlook. You may also load your own organizational logo to the form. The site is also protected. There is also an Android phone loaded application with the possibility to fill in the form offline by phone. This is seldom needed for simple food or water borne outbreak investigations, but is useful for e.g. weekly reporting forms from the field.
- A tip: We usually only ask the first name and two first letters of the family name to avoid uploading sensitive information. This protects the individuals. For the cohort, names of the participants are usually known, so it is only a matter of combining the list to the answers. An even safer option is to give every participant a separate code to use when filling in the questionnaire. We have had no problems in this respect.

Quick instructions to formulate the excel spreadsheet:

- More technical instructions may be found (in English) at: http://xlsform.org/ or https://opendatakit.org/use/xlsform/
- Compare carefully the excel spreadsheet and its questionnaire on the web by clicking different options and observing how the form changes https://ee.kobotoolbox.org/x/#iicYWqgG
- Excel spreadsheet has three sheets (you must not change the names of the sheets): ”survey”, ”choices” and ”settings”
- ”settings”: the name of the form that will be the main header (form_title) and technical name for the program (form_id), note, no special characters, no spaces.
- ”choices”: includes the alternatives for the main page (”survey”). Compare them carefully with the form on the web to observe the logic.
    - list_name: needs to be identical to ”survey” sheet “name” column
    - name: the code that will be printed to the summary spreadsheet (0=no, 1= yes, 9=do not know/do not remember etc.)
    - label: the option that is visible to the participants (have a look on the web form).
- ”survey”: the main form. One line is basically one question/comment/defining groups. The names of the columns are:
    - type: the type of the question, these need to be standardized and the same, the most common ones are note, text, select_one, select_multiple, datetime

  - name: the technical name of the variable (no special characters, no spaces, rather short one preferable). This same name needs to be found for the ”choices” sheet ”list name” variables which are the of form “select_one” or ”select_multiple”
  - hint: A hint for the participant, have a look on the web form
  - label: The label in the web form for this question
  - constraint, appearance, default, calculation: usually empty, please refer to further information
  - constraint_message: text which will appear if the participant does not give an answer for a compulsory question
  - required: true, if a compulsory question (*)
  - relevant: This is a very important field as it defines if this question is presented to the participant or not. It depends what the participant has answered to preceding questions. For example, if asked about the dessert (yes/no/dk) and the participant answers ”yes”, then a further question will appear on the quantity eaten. Therefore for the dessert1_q will be marked as for the relevant column: selected(${dessert1},’1’), ie. Only shown if answered ”yes”(=1) to dessert1 question.
  - read_only: If the variable is only information and no answer is required.
- You may test your form for the main logical errors here https://opendatakit.org/xlsform/ (browse, submit).

Practical instructions

- Go to the Kobo main website and create your own username and password (please note to use the correct login site) https://kf.kobotoolbox.org/forms//accounts/login/?next=/forms/
- After login, you are in the main page of your Kobo site
- To upload your new form → NEW → Upload an XLSForm → drag and drop the XLSForm here or click to browse
- To add the logo of your organization to the form: Click the form → SETTINGS → Media → +Add Document → Media Upload → Choose files → Upload ”form_logo.png”
- To collect the data after some submissions → DATA → Data → Downloads → XLS + XML values and headers (or choose yours)
- For troubleshooting, at the lower left corner of the main page there is help (?) button.
- As usual, best way to learn how to use the site is just by clicking and exploring.
